# A model for predicting *Xanthomonas arboricola* pv. *pruni* growth as a function of temperature

**DOI:** 10.1371/journal.pone.0177583

**Published:** 2017-05-11

**Authors:** Gerard Morales, Isidre Llorente, Emilio Montesinos, Concepció Moragrega

**Affiliations:** Institute of Food and Agricultural Technology-XaRTA-CIDSAV, University of Girona, Girona, Spain; Agricultural University of Athens, GREECE

## Abstract

A two-step modeling approach was used for predicting the effect of temperature on the growth of *Xanthomonas arboricola* pv. *pruni*, causal agent of bacterial spot disease of stone fruit. The *in vitro* growth of seven strains was monitored at temperatures from 5 to 35°C with a Bioscreen C system, and a calibrating equation was generated for converting optical densities to viable counts. In primary modeling, Baranyi, Buchanan, and modified Gompertz equations were fitted to viable count growth curves over the entire temperature range. The modified Gompertz model showed the best fit to the data, and it was selected to estimate the bacterial growth parameters at each temperature. Secondary modeling of maximum specific growth rate as a function of temperature was performed by using the Ratkowsky model and its variations. The modified Ratkowsky model showed the best goodness of fit to maximum specific growth rate estimates, and it was validated successfully for the seven strains at four additional temperatures. The model generated in this work will be used for predicting temperature-based *Xanthomonas arboricola* pv. *pruni* growth rate and derived potential daily doublings, and included as the inoculum potential component of a bacterial spot of stone fruit disease forecaster.

## Introduction

*Xanthomonas arboricola* pv. *pruni* [1, 2] is the causal agent of bacterial spot disease of stone fruit [3, 4], which is one of the most important diseases of *Prunus* species and their hybrids. Although it can affect all cultivated *Prunus*, the most severely affected hosts are peach, nectarine, apricot, and plum [5]. Recently, the disease has been reported on almond [6] and on cherry laurel [7, 8] in Europe. Disease symptoms include lesions on leaves, twigs, fruit, and stem cankers [9, 10]. Affected fruits are unmarketable, and infected trees may show severe defoliation with significant yield and quality losses [11].

The disease was first described in North America in 1903 [1], and currently, it is distributed throughout the major stone-fruit-producing areas worldwide [9]. The bacterium, considered a quarantine organism by the phytosanitary legislation of the European Union [12] and the European and Mediterranean Plant Protection Organization [3], has become locally established in several European countries, and it is currently spreading in many others, where local outbreaks have been reported [13, 14].

Disease control is based on preventive applications of copper-based bactericides and antibiotics [15]. Copper sprays applied early in the growing season have moderate efficacy [16] and have been associated with plant phytotoxicity [17]. Information on the most effective system for their application is limited, and copper sprays are not always applied at the appropriate time, when environmental conditions are favorable for bacterial infections. The disease is strongly influenced by the weather variables, mainly temperature, rain, and wetness [18, 19]. A disease forecasting model based on weather conditions could be used as a support system to guide pesticide applications [5] and increase disease control efficacy.

The pathogen population density is considered a critical parameter to forecast bacterial plant disease development [20–22]. Most forecasters of bacterial diseases are based on temperature-dependent bacterial multiplication in the presence of moisture to provide inoculum for infections, and on occurrence of weather conditions suitable for infections. Potential daily doublings of the associated pathogen are estimated from *in vitro* specific growth rates at different temperatures under optimal relative humidity conditions [20, 23, 24]. The optimum temperature and doubling times of *X*. *arboricola* pv. *pruni* were determined in a previous work [25], but no empirical models have been developed for predicting its growth as a function of temperature.

Predictive modeling has been widely used to estimate the growth of foodborne bacteria under different physical and chemical conditions [26–29] and to optimize the design and operation of bioreactors [30]. Primary and secondary models are used for predicting bacterial growth [28, 31]. Primary models describe the changes in population size over time under isothermal conditions [32]. Sigmoidal models, such as the modified Gompertz model [33], Baranyi model [30], and Buchanan model [34], can be used to fit bacterial growth to the three kinetic phases: the lag phase, exponential growth phase, and stationary phase. Secondary models are constructed to describe the growth parameters obtained from primary models as a function of independent variables, such as temperature [32].

*In vitro* bacterial growth can be expressed in terms of microbial density or optical density as an indirect measurement. Optical density measurements are less time consuming and allow for monitoring of bacterial population growth in real time. There are some limitations to directly fitting primary growth models to the optical density measurements [35, 36]. The use of calibration factors [24, 36] and calibration curves to recalculate the optical density data to viable count data [37, 38] have been proposed to correct the non-linearity of absorbance measurements.

The aim of this work was to develop a mathematical model for predicting *X*. *arboricola* pv. *pruni* growth *in vitro* as a function of temperature. Prior to growth curve modeling, the relationship between optical density and viable count data was analyzed, and a calibration curve was developed. Primary models were used to estimate the growth kinetic parameters, namely the maximum specific growth rate (μ_max_) and the lag time, under isothermal conditions within a temperature range from 5 to 35°C. Afterwards, aiming at the description of the estimated growth rate as a function of temperature, secondary models were developed and validated.

## Materials and methods

### Bacterial strains

Seven strains of *X*. *arboricola* pv. *pruni* selected from different host plant species and geographic region were used (Table 1). The strains were routinely grown at 27°C on Yeast-Peptone-Glucose Agar (YPGA) [16] and maintained at -70°C in YPG broth [16] supplemented with glycerol (20% wt/vol).

**Table 1:** *Xanthomonas arboricola* pv. *pruni* strains used in the study.

Strain ^y^	Host	Geographic region
CFBP 3894 ^z^	*Prunus salicina*	New Zealand
CFBP 3903	*Prunus domestica*	Italy
CFBP 5530	*Prunus persica*	Italy
CFBP 5563	*Prunus persica*	France
CFBP 5725	*Prunus persica*	EUA
IVIA 33	*Prunus amygdalus*	Spain
IVIA 3162–1	*Prunus amygdalus*	Spain

^y^ CFBP: Collection Frainçaise de Bactéries Phytopatogènes (Angers, France); IVIA: Instituto Valenciano de Investigaciones Agrarias (Moncada-Valencia, Spain).

^z^ Pathotype strain

### Relationship between optical density and viable cell count

The *in vitro* growth of *X*. *arboricola* pv. *pruni* was modeled from continuous absorbance measurements taken with an automated turbidimetric system (Bioscreen C, Labsystem, Helsinki, Finland). A calibration curve was generated to recalculate the optical density data to viable count data. The seven strains of *X*. *arboricola* pv. *pruni* listed in Table 1 were used to obtain the calibration curve. Bacterial suspensions in sterile distilled water were prepared individually for each strain from cultures grown on Luria-Bertani (LB) agar [39] for two days at 27°C. Suspensions were adjusted to 1–5 x 10^8^ CFU/ml, corresponding to an optical density at 600 nm of 0.5 [40]. LB was used instead of YPGA to reduce xanthan production. In order to evaluate a wider range of optical densities and bacterial concentrations, the bacterial suspensions were 10- and 100-fold diluted in sterile distilled water, and the initial concentrations of 10^6^, 10^7^, and 10^8^ CFU/ml were used. Honeycomb 100-well microplates were filled with 180 μl LB broth and inoculated with 20 μl of the corresponding bacterial suspension. Negative controls were inoculated with 20 μl sterile distilled water. Six wells were used per strain and concentration. The inoculated multiwell plates were incubated in the Bioscreen C system at 25°C for 60 h. Optical density measurements at 600 nm were performed every 60 min with 10 s of shaking prior to reading. At 1, 12, 24, 36, 48, and 60 h of incubation, after the optical density reading, 100 μl of the bacterial suspensions were removed from the corresponding wells for each strain and initial concentration, and appropriate 10-fold serial dilutions were plated in duplicate onto YPGA medium and incubated for three days at 27°C to determine the viable count. After removing the bacterial suspension, the wells were excluded from further analysis. The optical densities, corrected with the values from the negative controls, and corresponding log_10_-transformed data of the viable counts were used to fit the regression curves. The Beer-Lambert, quadratic [41], cubic [42], and logarithmic [43] equations were fitted to the data (equations 1, 2, 3, and 4, Table 2) using SPSS v. 23.0 software (IBM Corp., Armonk, NY). The *R-*squared, adjusted *R*-squared, and the number of parameters in the model were the criteria used to select the equation for the calibration curve.

**Table 2:** Equations of models used in the study.

Model	Equation ^z^	
Beer-Lambert	log_10_ N = a + b ∙ OD	(1)
Quadratic	log_10_ N = a + b ∙ OD + c ∙ OD^2^	(2)
Cubic	log_10_ N = a + b ∙ OD + c ∙ OD^2^ + d ∙ OD^3^	(3)
Logarithmic	log_10_ N = a + b ∙ ln OD	(4)
Baranyi	$\log_{10}N_{t}=\log_{10}N_{max}+\log_{10}\left(\frac{-1+exp(\mu_{max}∙lag)+exp(\mu_{max}∙t)}{exp(\mu_{max}∙t)}\right)-1+exp(\mu_{max}∙lag)∙10^{A}$	(5)
Buchanan	Lag phase: for t ≤ t_lag_, N_t_ = N_0_ Exponential growth phase: for t_lag_ < t < t_max_, N_t_ = N_0_ + μ(t − t_lag_) Stationary phase: for t ≥ t_max_, N_t_ = N_max_	(6)
Modified Gompertz	$\log_{10}N_{t}=\log_{10}N_{0}+A∙exp\{-exp[\frac{\mu_{max}∙e}{A}∙(lag-t)+1]\}$	(7)
Ratkowsky	μ_max_ = [b (T − T_min_)]^2^	(8)
Modified Ratkowsky	μ_max_ = (b (T − T_min_) ∙ {1 − exp[c (T − T_max_)]})^2^	(9)
Modified Ratkowsky	μ_max_ = [b (T − T_min_)]^2^ ∙ {1 − exp[c (T − T_max_)]}	(10)

^z^ A: logarithmic increase of bacterial population log_10_ (CFU/ml); e: exp(1); lag: lag time (h); N: cell concentration; N_0_ and N_max_: initial and final population densities, respectively (CFU/ml); N_t_: population density at time t (CFU/ml); OD: optical density; t: time (h) in logistic models; t_lag_: time to the end of lag phase (h); t_max_: time when the maximum population density is reached (h); T: temperature (°C); T_min_ and T_max_: minimum and maximum temperatures, respectively, at which the specific growth rate is zero; μ: specific growth rate in Buchanan model (h^-1^); μ_max_: maximum specific growth rate (h^-1^).

### Bacterial growth

Bacterial suspensions (1–5 x 10^8^ CFU/ml) of seven *X*. *arboricola* pv. *pruni* strains (Table 1) were prepared as described previously and 10-fold diluted in sterile distilled water. The wells of 100-well Honeycomb plates were filled with 180 μl LB broth and inoculated with 20 μl of a 1–5 x 10^7^ CFU/ml bacterial suspension, so that the initial bacterial concentration in each well was 1–5 x 10^6^ CFU/ml. Water activity (a_w_) of initial bacterial suspensions in LB broth ranged from 0.974 to 0.976, similar to LB broth (a_w_ = 0.975). Measurements of a_w_ were performed for all strains using a Novasina LabMaster-aw device (Novasina AG, Lachen, Switzerland). For each incubation temperature, three replicates of three wells were inoculated with each strain separately. Wells inoculated with 20 μl distilled water were used as blanks. The inoculated plates were incubated in the Bioscreen C system (Labsystem, Helsinki, Finland) at 15, 20, 25, 30, 33, 34 and 35°C for five days. Measurements were performed every 60 min with 10 s of shaking prior to the optical density reading at 600 nm. Incubations at 5 and 10°C were conducted in a growth chamber (MLR-350 Growth Cabinet, SANYO, Japan) for ten days and optical density measurements were performed twice a day by placing plates in the Bioscreen C system maintained at 15°C in a cold room to avoid problems with establishing temperature and optical density stability. The experiment was repeated twice.

The optical density values at each time point of inoculated wells were corrected with the optical densities of blanks. OD data at each time point of three wells in a replicate were averaged prior to data analysis. The calibration curve obtained previously was used to recalculate the optical density data to viable count data. Growth curves were obtained by plotting log_10_ (viable count) against the incubation time. Three growth curves were generated per strain and temperature in each of two independent experiments.

### Primary modeling of *X*. *arboricola* pv. *pruni* growth

*X*. *arboricola* pv. *pruni* growth *in vitro* at each temperature was modeled using primary models. Six viable cell count growth curves were used per strain to estimate the growth parameters at each temperature. A total of forty two growth curves were modelled per temperature. The Baranyi [30], Buchanan [34], and modified Gompertz [33] models (equations 5, 6, and 7, Table 2) were fitted to the growth curves under isothermal conditions by nonlinear regression using R (R Development Core Team 2015) package nlstools [44]. The model with the lowest residual sum of squares (RSS) was selected to estimate the maximum specific growth rate (μ_max_) and lag time for *X*. *arboricola* pv. *pruni* at each temperature. The doubling time (DT) was calculated as DT = ln 2 / μ_max_. The effects of strain, experiment, and temperature on the specific growth rate were determined using the general linear models (GLM) procedure after confirmation of normality and homoscedasticity, and mean comparison was performed with Tukey’s HSD test. The non-parametric test procedure for independent samples was used for determining the effects of experiment and strain on the lag time using the Mann-Whitney U and Kruskal-Wallis test, respectively. Arrhenius plot on the logarithm of the specific growth rate and reciprocal temperature was drawn and regions with significant linearity were detected by linear regression.

### Secondary modeling of *X*. *arboricola* pv. *pruni* growth

Secondary models were used to describe changes in the maximum specific growth rate as a function of temperature. The square root model Ratkowsky and its variations (equations 8, 9 and 10, Table 2) [28, 45, 46], were fitted to the averaged maximum specific growth rates of the *X*. *arboricola* pv. *pruni* strains derived from the primary models by nonlinear regression. The average of maximum specific growth rates over experiments and strains was used for secondary modeling to reduce data variability and improve curve fitting [47], since no differences were observed among strains and between experiments at any temperature (*P* > 0.057). The criteria for choosing the best model were the minimum RSS and the lowest number of parameters in the model. Furthermore, doubling times for *X*. *arboricola* pv. *pruni* at different temperatures reported in the literature [25] were back transformed to specific growth rates and the modified Ratkowsky equation 10 (Table 2) was fitted to the data as described previously. The optimum temperature for *X*. *arboricola* pv. *pruni* growth (at which the growth rate is maximum) was determined by equaling the first derivative of modified Ratkowsky model to zero.

### Validation of *X*. *arboricola* pv. *pruni* growth models

The model obtained for predicting the maximum specific growth rate of *X*. *arboricola* pv. *pruni* as a function of temperature was validated using data derived from four additional experiments at temperatures of 17, 22, 27, and 31°C for the seven strains separately (Table 1). Assays were performed as described previously. Briefly, three replicates of three wells per replicate filled with 180 ml LB were inoculated with 20 **μ**l 1–5 x 10^7^ CFU/ml suspensions of each strain and incubated at the corresponding temperature for three to five days in the Bioscreen C system (Labsystem, Helsinki, Finland). Optical density (600 nm) measurements were performed hourly and transformed to viable count values using the calibration curve obtained earlier. Averaged data of three wells in a replicate at each reading time were used in data analysis. A total of twenty-one growth curves were generated, three per strain and temperature. The modified Gompertz model (equation 7, Table 2) was used to estimate the observed maximum specific growth rate for each growth curve. The predicted specific growth rates at the new tested temperatures were calculated with the modified Ratkowsky model developed earlier. The correlation between observed and predicted data, and the performance indices of bias factor (Bf) and accuracy factor (Af) [48], were used for evaluating the performance of the constructed predictive models. A perfect consistency in a predictive model would have Bf = Af = 1. Bf values ranging from 0.9 to 1.05 are considered good, the range of 0.7–0.9 or 1.06–1.15 is considered acceptable, and either < 0.7 or > 1.15 is considered unacceptable [31]. Otherwise, the Af will increase by 0.1 to 0.15 for each predictive variable. In this study, the acceptable range for Af was less than 1.15 because only one environmental factor was employed.

## Results

### Relationship between optical density and viable cell count

**Fig 1:**
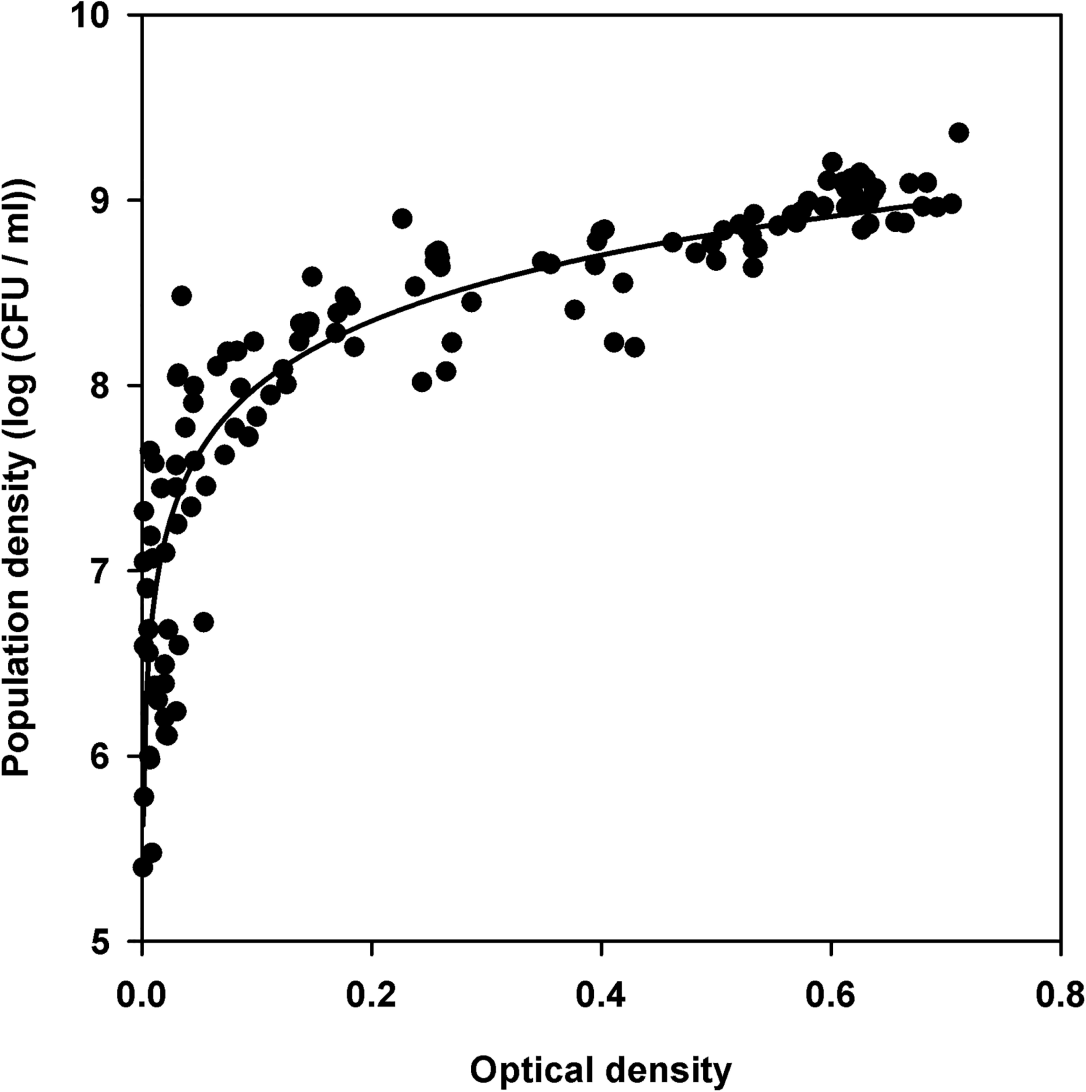
Relationship between population density and optical density at 600 nm for *X*. *arboricola* pv. *pruni*. Data from suspensions of seven strains incubated at 25°C were used. The curve generated by the logarithmic model is shown.

Individual data pairs were pooled to analyze the relationship between optical density and viable count in *X*. *arboricola* pv. *pruni*. In total, 126 data points were used to generate a unique calibration curve. Optical densities corrected with blank values ranged from 0.001 to 0.711 for bacterial densities from 5.40 to 9.36 log_10_ (CFU/ml) (Fig 1). A large increase in viable counts but low variation in optical density was observed at optical densities below 0.07, whereas increases in optical densities from 0.2 to 0.8 were related to viable count increases from 8 to 9.36 log_10_ CFU/ml (Fig 1). Beer-Lambert (linear), quadratic, cubic, and logarithmic models (equations 1, 2, 3 and 4, respectively, Table 2) were fitted to the data for the entire data range. The regression analysis results and parameter estimates are summarized in Table 3. The linear (equation 1) and quadratic (equation 2) models gave the poorest fits to the data, whereas the best fits to the data were achieved with the cubic (equation 3) and logarithmic (equation 4) models (Table 3). A model accepted statistically with as few parameters as possible was the criterion for selecting the logarithmic model for generating the calibration curve, for which the equation was *log*_*10*_ *N = 9*.*17 + 0*.*51 ln OD* (Fig 1), where *OD* is the optical density at 600 nm and *N* is the population density (CFU/ml). This equation was further used to transform optical densities to viable counts.

**Table 3:** Regression analysis between optical density at 600 nm and viable count for *X*. *arboricola* pv. *pruni* with different models.

Model ^x^	Model Summary ^y^					Parameter Estimate ^z^			
	*R*^*2*^	*R*^*2*^_*adj*_	*F*	df1	df2	a	b	c	d
Beer-Lambert (1)	0.660	0.657	240.57	1	124	7.20 (0.08)	3.14 (0.20)		
Logarithmic (2)	0.812	0.810	534.02	1	124	9.17 (0.06)	0.51 (0.02)		
Quadratic (3)	0.757	0.753	191.53	2	123	6.88 (0.08)	8.27 (0.75)	-7.95 (1.13)	
Cubic (4)	0.811	0.807	174.44	3	122	6.63 (0.08)	16.13 (1.49)	-40.01 (5.52)	32.17 (5.45)

^x^ Model equations are displayed in Table 2.

^y^ All model *F* values were highly significant (*P* < 0.0001). df1: regression effective degrees of freedom; df2: residual effective degrees of freedom.

^z^ Models were fitted to 126 data points using linear regression analysis. Standard errors are reported in parentheses.

### Modeling bacterial growth

Optical densities obtained from the Bioscreen C system were transformed to viable counts using the calibration curve generated in this study, as described above. In total, 189 viable count growth curves were obtained per experiment, corresponding to three replicates of seven strains per temperature. All *X*. *arboricola* pv. *pruni* strains were able to grow *in vitro* at temperatures from above 5 to 34°C. No growth was observed for the first five days of incubation at 5°C and longer incubation durations were needed to detect bacterial growth at this temperature, which could be considered the minimum for *X*. *arboricola* pv. *pruni* multiplication. Growth at 35°C was variable, depending on the strain. The type strain CFBP 3894 was unable to grow at 35°C, and only 24 out of 42 growth curves for the other strains showed growth at 35°C and were included in the analysis.

**Fig 2:**
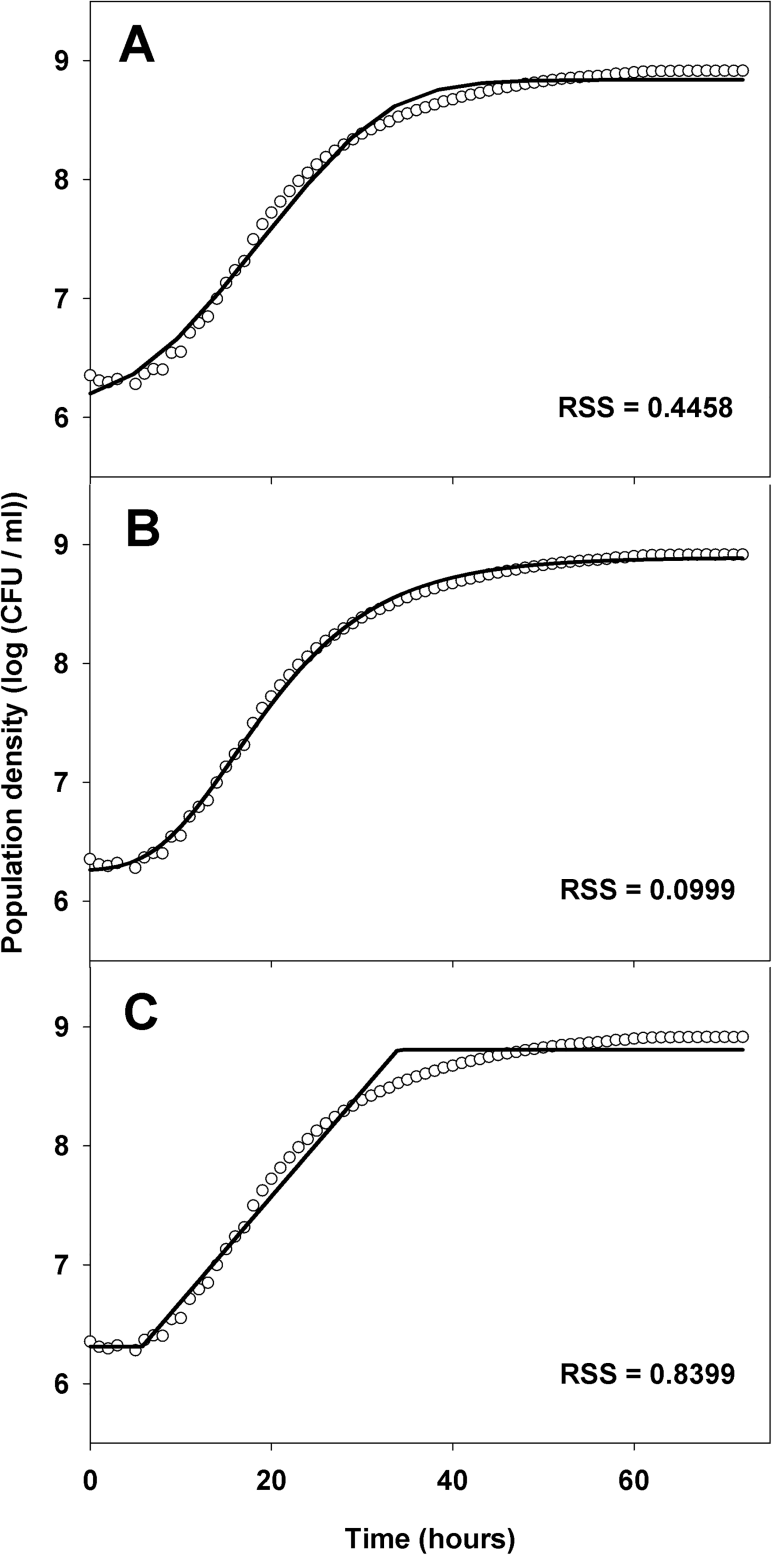
Primary model fitting to one of the six experimental growth curves for *X*. *arboricola* pv. *pruni* strain CFBP 5530 at 25°C. (A) Baranyi, (B) Gompertz modified, and (C) Buchanan models were fitted to experimental data. The residual sum of squares (RSS) for each model is reported.

The Baranyi, Buchanan, and modified Gompertz models (equations 5, 6, and 7, Table 2) were fitted to the 189 log_10_-transformed viable count growth curves of each experiment by nonlinear regression to estimate the growth parameters for each *X*. *arboricola* pv. *pruni* strain and temperature replicate. Model fitting to one growth curve of *X*. *arboricola* pv. *pruni* strain CFBP 5530 at 25°C is shown in Fig 2, where the RSS values of the Baranyi, modified Gompertz and Buchanan models are shown and representative of all datasets. The modified Gompertz model (equation 7, Table 2) showed the best fit to the data in all data sets, with the lowest RSS (data not shown), and was selected to estimate the maximum specific growth rate and lag time of *X*. *arboricola* pv. *pruni* strains at each temperature. The *F-*test results indicated no significant differences in the maximum specific growth rates between the two independent experiments (*P* = 0.1848). Similarly, the maximum specific growth rates were not significantly different among strains (*P* > 0.057) at any temperature. Therefore, the maximum specific growth rates were pooled over experiments and strains and averaged data were used in secondary modeling.

**Fig 3:**
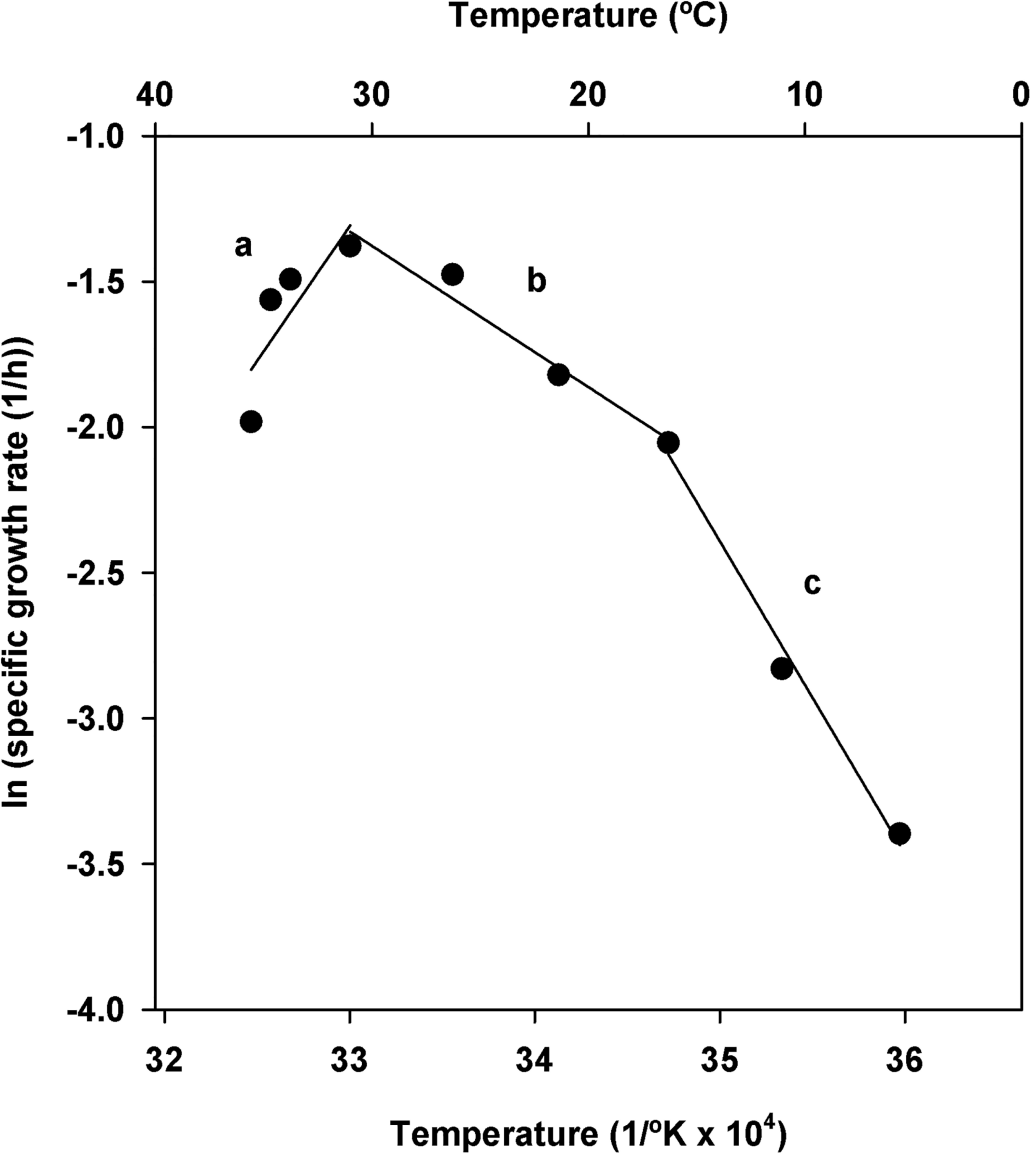
Arrhenius plot of the maximum specific growth rates for *X*. *arboricola* pv. *pruni*. Lines show three linear regions: (a) *ln (μ*_*max*_*)* = *0*.*92T – 31*.*81* (*R*^*2*^ *=* 0.66); (b) *ln (μ*_*max*_*) =* -*0*.*41T + 12*.*35* (*R*^*2*^ *=* 0.97); and (c) *ln (μ*_*max*_*) =* -*1*.*08T + 35*.*18* (*R*^*2*^ *=* 0.99). Where *T* is 1/°K x 10^4^.

The means of the maximum specific growth rate and doubling time at each temperature are presented in Table 4. A significant effect of temperature on the maximum specific growth rate (*P* < 0.001) was observed, and differences among temperatures were detected according to Tukey’s HSD test (Table 4). The maximum specific growth rate of *X*. *arboricola* pv. *pruni* increased progressively when the temperature increased from 5 to 30°C, and decreased at temperatures above 30°C. The highest growth rates were obtained at temperatures from 25 to 33°C, whereas the significantly lowest growth rates were observed at 5 and 10°C (Table 4). The Arrhenius plot in Fig 3 shows the relationship between the logarithm of the maximum specific growth rate and temperature. The curve is characterized by a continuously changing slope between the minimum (5 to 15°C), medium (15 to 30°C), and maximum (30 to 35°C) temperatures. Regions of linearity can be observed corresponding to these three intervals of temperature, but not over the entire range. Linear equations obtained for each region are displayed (Fig 3).

**Table 4:** Growth parameters and corresponding standard error for *X*. *arboricola* pv. *pruni* at different temperatures (T) estimated by the modified Gompertz model.

T (°C)	Maximum specific growth rate (h^-1^) ^x^		Doubling time (h)	Lag time (h) ^x^
5	0.033 ± 0.002	d ^y^	20.69 ± 1.20	92.27 ± 4.33	
10	0.059 ± 0.004	d	11.74 ± 0.76	37.22 ± 2.19	
15	0.128 ± 0.006	c	5.41 ± 0.26	26.29 ± 1.57	
20	0.162 ± 0.006	c	4.28 ± 0.16	5.67 ± 0.62	
25	0.228 ± 0.010	ab	3.03 ± 0.14	6.58 ± 0.67	
30	0.252 ± 0.009	a	2.75 ± 0.10	2.49 ± 0.34	
33	0.225 ± 0.006	ab	2.86 ± 0.08	2.38 ± 0.37	
34	0.209 ± 0.007	b	3.08 ± 0.11	7.72 ± 1.29	
35 ^z^	0.138 ± 0.006	c	3.31 ± 0.24	33.68 ± 4.98	

^x^ Values are the mean of parameter estimates from the modified Gompertz equations obtained for 42 growth curves at each temperature, corresponding to seven strains and three replicates per strain in two independent experiments.

^y^ Means within the same column followed by the same letter do not differ significantly (*P* = 0.05) according to the Tukey’s HDS mean comparison test.

^z^ Growth at 35°C was variable. Only data from strains that were able to grow at 35°C were included.

The negative lag times estimated from the modified Gompertz model were assumed to be 0. No significant differences (*P =* 0.072) were observed in lag times between experiments at all temperatures, except for 5, 15, and 25°C, according to the Mann-Whitney U test. The results from the Kruskal-Wallis test indicated no significant differences in lag time among strains at all temperatures (*P =* 0.079), except for 35°C (*P =* 0.017). Mean lag time values of seven strains at each temperature are presented in Table 4. The mean of the lag time decreased when temperature increased from 5°C to 33°C. Above 33°C the lag time increased to a maximum at 35°C (Table 4).

**Fig 4:**
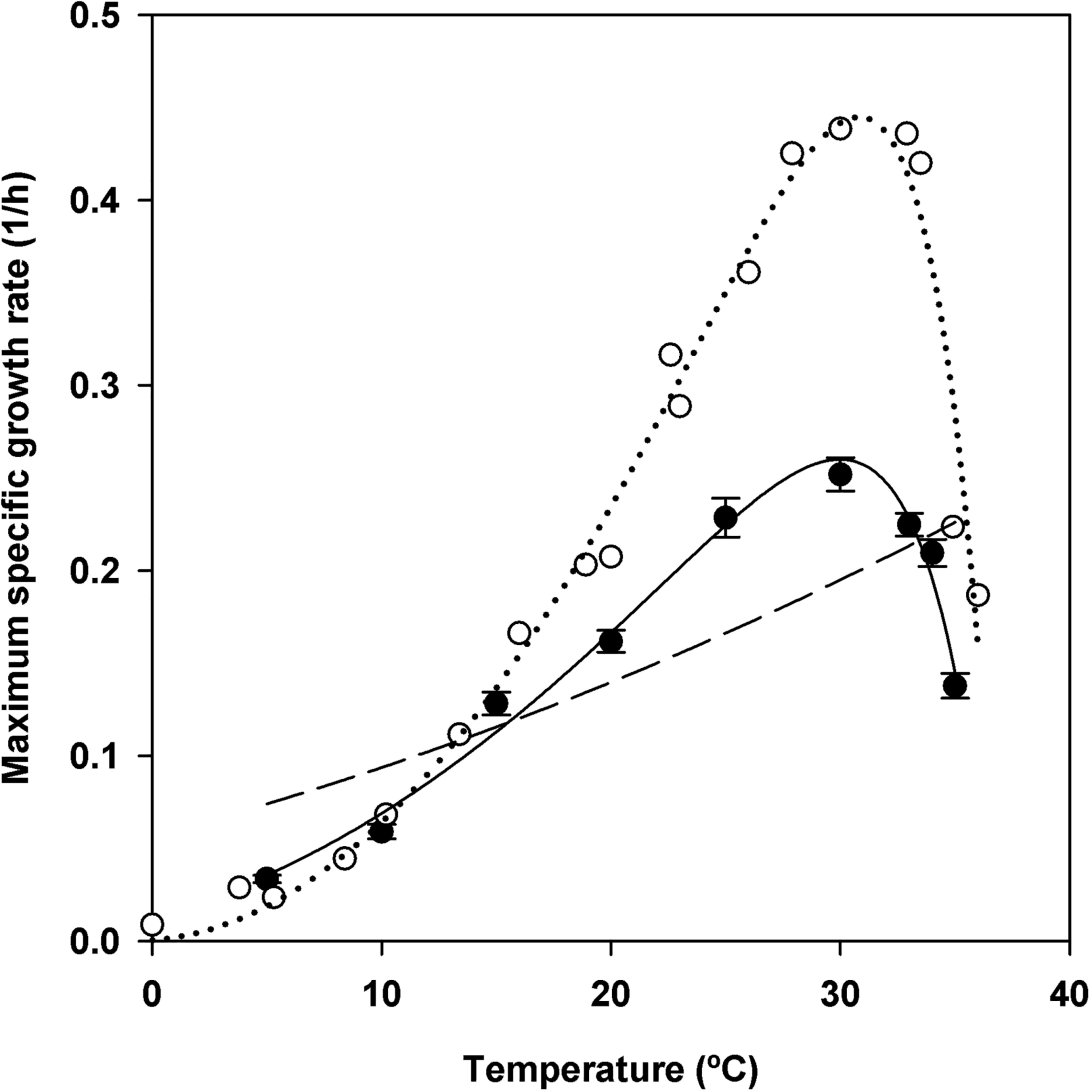
Model fitting to the maximum specific growth rate for *X*. *arboricola* pv. *pruni* as a function of temperature. Values of the maximum specific growth rate (black symbols) are the mean of two experiments, seven strains and three replicates per strain. Error bars are the standard errors. Modified Ratkowsky models are coincident and represented with continuous line; dashed line represents the Ratkowsky model. The modified Ratkowsky model (equation 10) fitting to the growth rate data from the literature (white symbols) [25] is shown in dotted line.

In the second step of modeling, the Ratkowsky equation and its modifications (equations 8, 9 and 10, Table 2), were fitted to modified Gompertz estimates of the specific growth rates at the tested temperatures. Non-linear regressions were based on averaged data for the specific growth rate for each temperature presented in Table 4. The parameter estimates and statistical analysis are presented in Table 5. The Ratkowsky model (equation 8) did not fit well to the data (Table 5 and Fig 4). The modified Ratkowsky equations 9 and 10 described bacterial growth over the entire temperature range and were found to be more appropriate (Table 5). The minimum and maximum temperatures estimated from each model are presented in Table 5. The maximum temperatures from the modified Ratkowsky models were close to 35°C, the highest temperature at which some strains of *X*. *arboricola* pv. *pruni* were able to growth, whereas the minimum temperature estimates were lower than the minimal temperature tested in this work and do not have biological meaning (Table 5). The modified Ratkowsky equation 10 had the lowest RSS and the highest adjusted *R*-squared (Table 5). Therefore, this model was chosen for predicting the *in vitro* specific growth rate of *X*. *arboricola* pv. *pruni* as a function of temperature. The optimal temperature for *X*. *arboricola* pv. *pruni* growth *in vitro* derived from this equation was 29.97°C, with a specific growth rate of 0.26 h^-1^ and a doubling time of 2.66 h. These values agreed with experimental data (Table 4).

**Table 5:** Parameter estimation and statistical evaluation for the secondary models describing the maximum specific growth rate for *X*. *arboricola* pv. *pruni* as a function of temperature.

Model parameter and statistics	Maximum specific growth rate models ^*y*^		
	Ratkowsky (8)	Modified Ratkowsky (9)	Modified Ratkowsky (10)
b	0.007 (0.002) ^z^	0.014 (0.001)	0.015 (0.002)
c	-	0.332 (0.087)	0.270 (0.075)
Tmin	-35.15 (21.54)	-8.27 (2.78)	-7.77 (2.64)
Tmax	-	37.86 (0.71)	36.67 (0.41)
RSS	0.0187	0.0008	0.0007
*R*^*2*^_*adj*_	0.5512	0.9727	0.9766

^y^ Equations of Ratkowsky model and its variations are listed in Table 2. Equation number is shown in parentheses.

^z^ Standard error of estimates are reported in parentheses.

In addition, the modified Ratkowsky equation 10 (Table 2) fitted well to specific growth rates at different temperatures (*T*) reported for *X*. *arboricola* pv. *pruni* in the literature [25] (Fig 4). The equation obtained was: μ_*max*_ *= (0*.0233 * (T—(-0.*8714)))*^*2*^ * (1—exp(0.2801 * (T—36.*8614)))* (RSS = 0.0093 and adjusted *R*-squared = 0.9717). The optimal temperature for *X*. *arboricola* pv. *pruni* growth estimated from this equation was 30.82°C.

### Model validation

The predictive model based on the modified Ratkowsky equation 10 (Table 5) was evaluated at four new temperatures (17, 22, 27 and 31°C) for the seven strains. No significant differences (*P <* 0.001) were observed among strains in the maximum specific growth rate derived from the modified Gompertz model at each temperature. Table 6 shows the averaged maximum specific growth rate of seven strains and three replicates per strain and those predicted with the secondary model equations generated in this work. The correlation between the predicted and observed maximum specific growth rates, as well as the indices of bias and accuracy were determined. A significant correlation between the predicted and observed specific growth rates was obtained for the modified Ratkowsky model (*P* = 0.026), with a Pearson coefficient *r* = 0.974. Bias (0.93) and accuracy factors (1.08) were also in the good range.

**Table 6:** Observed and predicted maximum specific growth rate for *X*. *arboricola* pv. *pruni* at temperatures tested in model validation.

Temperature (°C)	Maximum specific growth rate (h^-1^)	
	Observed ^y^	Predicted ^z^
17	0.137 ± 0.009	0.133
22	0.198 ± 0.008	0.190
27	0.257 ± 0.011	0.245
31	0.313 ± 0.015	0.257

^y^ Values are the mean of parameter estimates from the modified Gompertz equations obtained for 21 growth curves at each temperature, corresponding to seven strains and three replicates per strain.

^z^ Maximum specific growth rate predicted by the modified Ratkowsky equation: μ_*max*_ *= (0*.015 * (T—(-7.*77)))*^*2*^ * (1—exp(0.270 * (T—36.*67)))*.

## Discussion

Understanding the growth parameters of *X*. *arboricola pv*. *pruni*, as well as their variation with temperature, is essential and the first step in the development of a model for predicting infections on host plants. Doubling times and daily potential doublings, calculated from specific growth rates at different temperatures, are often included in bacterial plant disease forecasting models [21, 23, 24]. Growth studies on plant pathogenic bacteria are generally based on the manual quantification of viable counts or optical density measurements at discrete time intervals, and parameter estimation is performed directly on either partly or fully experimental growth curves [20, 21, 25]. Predictive modeling of bacterial growth is an area in food microbiology where the effect of environmental factors on bacterial growth are quantified and modeled with mathematical equations [31, 48]. In our work, predictive microbiology was used to describe the response of *X*. *arboricola pv*. *pruni* to temperature. The results demonstrated that the *in vitro* growth of *X*. *arboricola pv*. *pruni* can be modeled and that primary and secondary models can be used for predicting the specific growth rate as a function of temperature. To our knowledge this is the first time that predictive microbiology is used for modeling the growth of plant pathogenic bacteria, and a similar approach could be used in other bacterial species.

The development of models in predictive microbiology requires large quantities of data obtained from growth curves. The classical method for constructing bacterial growth curves, based on plate counting of culture samples taken at several time intervals, is laborious and time consuming, whereas optical density measurements are rapid and nondestructive and, if automated, provide real-time growth curves. Bioscreen C, an automated turbidimetric system, is an adequate system for obtaining data for predictive microbiology, and made it possible to generate and model a large number of growth curves for *X*. *arboricola* pv. *pruni* incubated at a wide range of temperatures. However, when modeling optical density growth curves, the fitted parameters are different from the parameters derived from viable counts [36, 37], and calibration factors are needed [26]. In the present work, a calibration curve to convert optical densities to viable counts was obtained for *X*. *arboricola* pv. *pruni* in order to overcome the limitations of the direct modeling of optical density growth curves [36]. Among the four equations evaluated, the Beer–Lambert equation showed a low goodness of fit to the entire data range, from 10^5^ to 10^9^ CFU/ml, as expected, because there is only a narrow range where the relationship between turbidity values and bacterial concentrations is linear [49]. The logarithmic transformation of the optical density and viable count data, which resulted in a linear relationship, produced the best fit to the data. This equation was used to recalculate the optical density data to viable counts, prior to the primary modeling of *X*. *arboricola* pv. *pruni* growth. A similar calibrating relation was used modeling *Bacillus cereus* growth [50].

Primary models describe the evolution of the bacterial population over time. The modified Gompertz equation [33], the model of Baranyi and Roberts [30], and the Buchanan model [34] are among the most widely used and were chosen for fitting to the *X*. *arboricola* pv. *pruni* growth curves. At all temperatures, the modified Gompertz model showed the best goodness of fit, with the lowest RSS. Consequently, it was selected to estimate the kinetic parameters of *X*. *arboricola* pv. *pruni* at each temperature. The selection of an adequate equation is especially important when practical applications are derived from the model estimates, because their values may differ depending on the predictive model used [33]. A significant effect of temperature on the maximum specific growth rate of *X*. *arboricola* pv. *pruni* was observed. The highest specific growth rates and the lowest doubling times were obtained at temperatures from 25 to 33°C, with a maximum at 30°C. The specific growth rates were lower below and above this range and minimal at 5 and 10°C. Therefore, the suboptimal temperatures for *X*. *arboricola* pv. *pruni* growth can be established as from 15 to 20°C. The specific growth rate at 34°C was higher than at 20°C, but only 50% of replicates for six out of the seven strains were able to grow at 35°C. The *in vitro* growth of *X*. *arboricola* pv. *pruni* had been previously reported at 35 and 36°C [25], with doubling times similar to those obtained at 35°C in our study. The optimal temperatures for the growth of *X*. *arboricola* pv. *pruni* are similar in both studies and similar to that of *X*. *campestris* pv. *vesicatoria* [21], but they are higher than those determined for other plant pathogenic bacteria, such as *Erwinia amylovora* [20] and *Pseudomonas syringae* [25].

In secondary modeling, the Ratkowsky model only modeled the maximum specific growth rate below the optimal growth temperature and it was dismissed. The modified Ratkowsky models fitted the data well and were proved to be reliable in predicting the effect of temperature on the specific growth rate over the entire temperature range. Finally, the modified Ratkowsky equation 10 was selected because when extrapolating at temperatures above the maximum for growth, no positive specific growth rates are predicted [28]. This secondary model was successfully validated at four new temperatures for the seven bacterial strains. Additionally, the modified Ratkowsky model also fitted well to the specific growth rate data derived from a previous study [25], and similar estimates were obtained for a, b and Tmax parameters although higher specific growth rates were reported. Differences in growth kinetics have been reported among strains of foodborne pathogens such as *E*. *coli* [51]. Similarly, the differences in the growth rate obtained in the present work and those reported by Young et al. [25] may be attributed to strain biological variability. Young et al. [25] studies were based on a group of strains recovered from infected *Prunus sp*. samples in New Zealand (NZ), whereas our work was performed with strains recovered from host species in Europe (five strains), USA (one strain) and NZ (one strain). The strain CFBP 3894 used in our work, recovered from *Prunus salicina* in NZ, had not been tested in the previous work. On the other hand, composition of growth media can also affect the bacterial growth, since bacteria adapt their growth to nutrient availability. Not only differences in growth rate of *X*. *campestris* pv. *campestris* were observed depending on the growth medium [52], but also biofilm formation and bacterial aggregation was related to the media composition and particularly to the calcium content in the medium. Similarly, the differences in the growth rate of *X*. *arboricola* pv. *pruni* found in our work could be partially attributed to differences in the growth medium composition. Under the restricted growth conditions used in the two experiments, no nutrients were added during the incubation period, the type of nutrients and their concentration would limit the bacterial growth. Sezonov et al. [53] found that the steady-state growth of *E*. *coli* in LB broth ended early, followed by an extended period during which the growth rate decreased gradually, due to the lack of utilizable carbon sources. Finally, experimental and methodological aspects concerning starting bacterial densities or the physiological state of initial cells, could also explain in part the above mentioned differences in *X*. *arboricola* pv. *pruni* growth rates found in the two studies. However, the optimal temperature for *X*. *arboricola* pv. *pruni* growth was estimated to be 30°C by both equations.

Lag phase has been estimated by applying the Gompertz equation to OD growth curves with starting inoculum level sufficiently high to produce an initial OD above the detection threshold of the turbidimeters used [37]. However, lag phase modeling based on a narrow range of initial high bacterial concentrations, can lead to poor estimates when using empirical models such as the modified Gompertz. The modified Gompertz model may produce negative lag times that have no biological meaning [36] at temperatures close to the optimal temperature, when bacterial cultures grow without a lag phase. High variability of lag time was observed in our work at temperatures 5, 15 and 25°C. Variability in lag phase has been consistently reported in the literature; thus, poor estimates are often obtained for this parameter [54]. In fact, many parameters can affect the lag phase including, temperature history and the physiological state of initial cells, as well as the starting bacterial density. The lag phase allows time for the adaptation measures required for bacterial cells to begin to exploit new environmental conditions, and genes involved in translation, protein synthesis, cell polarity, cell division, and cell cycle control are induced [55]. Various factors may influence the lag phase apart from temperature [54]. A better understanding of the lag phase and the physiological factors that affect it is needed in order to use this parameter for epidemiological or disease forecasting purposes.

Previous studies reported that at least three successive rainy days with temperatures between 14 and 19°C were necessary for primary infections of *X*. *arboricola* pv. *pruni* on peach trees in Italy [18] and that a mean temperature of 12°C was sufficient to produce infections in France [56]. These temperatures are suboptimal for *X*. *arboricola* pv. *pruni* growth. In fact, daily mean temperatures do not reflect the real temperature over the entire day and short periods of temperatures above 15°C may be sufficient for the bacterial population to increase to potentially infective concentrations. Additionally, the leaf temperature of many plants are 5 to 10°C above the air temperature in direct sunlight [25], so temperatures on and in leaves may be more favorable for pathogen multiplication. Otherwise, primary infections on peach are observed from May to mid-July in Italy [18], when daily fluctuating temperatures may reach the optimal range for *X*. *arboricola* pv. *pruni* multiplication. The disease incubation period has been reported to be between 7 and 25 days in warm and cold weather, respectively; suggesting similar effects of temperature on *X*. *arboricola* pv. *pruni* multiplication in host plant tissues.

*X*. *arboricola* pv. *pruni* is an aerial plant pathogenic bacterium that can survive and multiply on the surface of host organs as epiphyte. Population densities of 1.3 x 10^2^ to 2.1 x 10^4^ CFU/g in peach and 3.2 x 10^3^ to 1.5 x 10^6^ CFU/g in plum were recovered during January, February and March [57], which may act as the inoculum reservoir for spring infections. Studies on pathogen epidemiology revealed that the relative humidity and especially water status of host plants are key parameters, as well as temperature, for *X*. *arboricola* pv. *pruni* multiplication and infections [58, 59]. Wetness is also required for *X*. *arboricola* pv. *pruni* infections on host tissues [56, 58, 59]. Preliminary studies on *X*. *arboricola* pv. *pruni* growth on the surface of peach leaves at optimal temperature and under different relative humidity conditions conducted in our laboratory demonstrated that epiphytic pathogen populations under low relative humidity rapidly declined, whereas they were able to colonize the leaf surface under wetness conditions (unpublished data).

The temperature-based model for predicting *X*. *arboricola* pv. *pruni* growth rate developed in this manuscript will be used for determining the potential bacterial multiplication as a first component of a bacterial spot of stone fruit disease forecasting model, in a similar approach as Maryblight [22], Cougarblight [24], and Billing’s integrated system [60] forecasting models for fire blight of apple and pear caused by *Erwinia amylovora*. Further studies on the effect of temperature on *X*. *arboricola* pv. *pruni* growth *in vivo* and on the effects of weather conditions on infections and disease development will be undertaken to develop infection models to be included in the disease forecaster.

*X*. *arboricola* pv *pruni* is a quarantine pathogen in the EU, so the practical applications of the disease forecaster are twofold: i) field assessment of disease spread and outbreaks based on infection risk prediction (specially for disease surveillance in regions were the disease is not stablished but some outbreaks have been detected), and ii) rational timing of products for disease control (bactericides), applied only when conditions are favorable for bacterial multiplication and infection.

## Conclusions

Predictive modeling of bacterial growth based on optical density measurements, widely used in food microbiology area, has been successfully applied for the plant pathogenic bacteria *X*. *arboricola* pv. *pruni*.

This work demonstrates that the effects of temperature on *X*. *arboricola* pv. *pruni* growth can be predicted. The model generated in this work has been successfully validated and will be used for predicting temperature-based *Xanthomonas arboricola* pv. *pruni* growth rate and derived potential daily doublings, and included as the inoculum potential component of a bacterial spot of stone fruit disease forecaster.
